# Hybridization-based capture of pathogen mRNA enables paired host-pathogen transcriptional analysis

**DOI:** 10.1038/s41598-019-55633-6

**Published:** 2019-12-17

**Authors:** Viktoria Betin, Cristina Penaranda, Nirmalya Bandyopadhyay, Rui Yang, Angela Abitua, Roby P. Bhattacharyya, Amy Fan, Roi Avraham, Jonathan Livny, Noam Shoresh, Deborah T. Hung

**Affiliations:** 1 Infectious Disease and Microbiome Program, Broad Institute of Harvard and MIT, 415 Main Street, Cambridge, MA 02142 USA; 2 Department of Molecular and Cellular Biology, Harvard University, 52 Oxford St, Cambridge, MA 02138 USA; 3 Department of Molecular Biology and Center for Computational and Integrative Biology, Massachusetts General Hospital, 185 Cambridge Street, Boston, MA 02114 USA; 4 Department of Genetics, Harvard Medical School, 77 Avenue Louis Pasteur, Boston, MA 02115 USA; 5 Infectious Diseases Division, Department of Medicine, Massachusetts General Hospital, 55 Fruit St, Boston, MA 02114 USA; 6 Present Address: Department of Biological Regulation, Weizmann Institute of Science, Rehovot, Israel

**Keywords:** Bacterial genomics, Bacterial host response

## Abstract

Dual transcriptional profiling of host and bacteria during infection is challenging due to the low abundance of bacterial mRNA. We report Pathogen Hybrid Capture (PatH-Cap), a method to enrich for bacterial mRNA and deplete bacterial rRNA simultaneously from dual RNA-seq libraries using transcriptome-specific probes. By addressing both the differential RNA content of the host relative to the infecting bacterium and the overwhelming abundance of uninformative structural RNAs (rRNA, tRNA) of both species in a single step, this approach enables analysis of very low-input RNA samples. By sequencing libraries before (pre-PatH-Cap) and after (post-PatH-Cap) enrichment, we achieve dual transcriptional profiling of host and bacteria, respectively, from the same sample. Importantly, enrichment preserves relative transcript abundance and increases the number of unique bacterial transcripts per gene in post-PatH-Cap libraries compared to pre-PatH-Cap libraries at the same sequencing depth, thereby decreasing the sequencing depth required to fully capture the transcriptional profile of the infecting bacteria. We demonstrate that PatH-Cap enables the study of low-input samples including single eukaryotic cells infected by 1–3 *Pseudomonas aeruginosa* bacteria and paired host-pathogen temporal gene expression analysis of *Mycobacterium tuberculosis* infecting macrophages. PatH-Cap can be applied to the study of a range of pathogens and microbial species, and more generally, to lowly-abundant species in mixed populations.

## Introduction

Simultaneous profiling of host and pathogen transcriptomes using dual RNA-seq is a powerful tool to study the complex interactions that occur during infection^1^. Dual RNA-seq has provided valuable insights into the host-pathogen dynamics, revealing important bacterial responses to the intracellular host environment such as changes in bacterial metabolism^2^ and iron utilization^3^. It has also elucidated host adaptations after perturbation by invading bacteria, such as macrophage polarization^4^ and inflammatory responses^5^. However, accurate bacterial gene expression profiling is currently a major limitation in dual RNA-seq studies.

Three central challenges have limited the use of dual RNA-seq to characterize host-pathogen interactions during bacterial infection. First, the amount of total RNA isolated from infected cells and tissues can be very low, especially in single eukaryotic cells. Second, bacterial cells have ~100-fold less total RNA than eukaryotic cells^6^ resulting in a large disparity between the number of bacteria- and host-derived transcripts within infected eukaryotic cells. Third, mRNAs represent only ~5% of total bacterial RNA, as non-coding structural RNAs, such as rRNAs, make up >95% of bacterial transcriptomes^6^. Thus, bacterial mRNA represents only ~0.05% of the total RNA in a eukaryotic cell infected with a single bacterium^6^. Together, these challenges make profiling bacterial gene expression during infection difficult and have limited the models of infection that can be studied using dual RNA-seq. Such models have either required extremely large experimental set-ups to obtain sufficient total RNA^7^, a multiplicity of infection that greatly exceeds that of physiological infection to increase the bacterial to host RNA ratio^8^, and/or very high sequencing depths per sample to capture the rare bacterial mRNAs. These challenges have severely limited the applicability and scale of dual RNA-seq studies^1^.

Several methods have been developed to enrich for the small percentage of mRNA from total RNA. Eukaryotic RNA-seq library construction methods selectively capture mRNA transcripts by leveraging their poly-adenylated tail; however, bacterial mRNAs lack polyadenylation and are not captured by these methods. Instead, non-specific capture of all RNAs is necessary to ensure bacterial mRNA inclusion in dual host-pathogen RNA-seq libraries. Depletion of bacterial and mammalian rRNA can then be achieved using commercially available kits. However, these depletion methods require relatively large quantities of total RNA input (10–100 ng), limiting their use for low input samples. Moreover, even after rRNA depletion, the vastly more abundant host mRNAs and non-coding RNAs dwarf the bacterial mRNAs, thus driving the high depth and costly sequencing required to capture the bacterial transcriptional program.

A complementary approach to depletion of unwanted RNA species is positive enrichment for desired RNAs through solution hybridization selection^9–12^, a flexible, scalable and efficient method for capture of low abundance sequencing targets. Positive enrichment methods have been shown to outperform depletion methods, achieving greater transcriptome coverage and accuracy of bacterial gene expression quantification at a lower sequencing depth^13^. Peterson *et al*. recently used this strategy to enrich for bacteria-derived targets from dual RNA-seq libraries constructed from infected cells in a method termed Path-seq^7^. However, because Path-seq enriches for all transcribed regions of the bacterial genome, including structural RNAs (rRNA and tRNAs), it requires an additional depletion step to eliminate the bacterial rRNA prior to library construction. This depletion step results in considerable loss of material, requiring a high starting RNA input and, thus, cannot be applied to small populations or single infected eukaryotic cells. To date, no method exists to perform transcriptional analysis of low input samples without cost-prohibitive sequencing, which currently constrains applications of dual RNA-seq.

**Figure 1:**
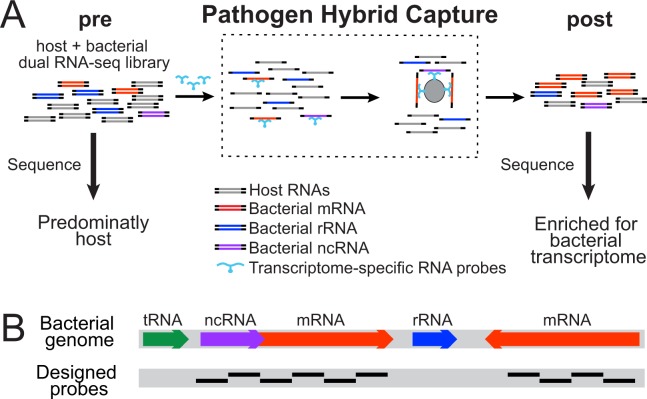
Pathogen Hybrid Capture selection method and probe design. (**A**) Pathogen Hybrid Capture (PatH-Cap) is applied to host and bacterial dual RNA-seq libraries to enrich for the bacterial transcriptome-derived templates. Pre-PatH-Cap libraries are incubated with bacterial transcriptome-specific biotinylated RNA probes that are used to pull out their complementary DNA template targets with streptavidin coated beads to yield post-PatH-Cap libraries. (**B**) Probes are designed as 100-mer sequences that tile along desired bacterial sequences (coding mRNAs and annotated noncoding RNAs (ncRNA)); rRNA and tRNA sequences are excluded.

To address this need, we developed a strategy, termed Pathogen Hybrid Capture (PatH-Cap). Because it simultaneously enriches for bacterial mRNA from the highly abundant host RNA and excludes bacterial structural RNA (rRNAs and tRNAs) in a single step, PatH-Cap can be successfully applied to libraries made from extremely low-input samples, even single infected cells (Fig. 1A). Enrichment of dual RNA-seq libraries is achieved through solution hybridization selection with bacterial transcriptome-specific probes. By sequencing standard dual RNA-seq libraries before enrichment (pre-PatH-Cap), to profile the host transcriptome, and after enrichment (post-PatH-Cap), to profile the pathogen transcriptome, we achieve dual transcriptional profiling of host and pathogen from the same sample, thereby facilitating the integrated analysis of their transcriptional responses. Importantly, enrichment increases the number of unique transcripts per gene in post-PatH-Cap libraries compared to pre-PatH-Cap libraries at the same sequencing depth; thus, to attain sufficient unique transcripts to allow for confident analysis of the bacterial transcriptome, lower sequencing depth is required. We demonstrate that PatH-Cap enables the study of single eukaryotic cells infected by 1–3 bacteria (epithelial cells infected by *Pseudomonas aeruginosa* PAO1) and paired analysis of host and bacteria over time (a temporal analysis of macrophages infected by *Mycobacterium tuberculosis* H37Rv). This enrichment strategy has the potential to be broadly applicable to the study of lowly-abundant species in mixed populations beyond host-pathogen interactions, including non-pathogenic bacteria as well as microbiome communities.

## Results

### PatH-Cap probe design and selection method

To develop PatH-Cap, a positive selection strategy to enrich for bacterial mRNA and, at the same time, deplete bacterial rRNA from dual RNA-seq libraries comprising a majority of host and bacterial rRNA, we designed probe-sets to selectively capture desired bacterial sequences. Our probe-sets included mRNAs and annotated noncoding RNAs (ncRNAs) sequences and excluded bacterial rRNA and tRNA sequences. Probe-sets consisted of 100-bp sequences tiled along desired bacterial regions (Fig. 1B). We designed a *M*. *tuberculosis* probe-set comprising 38,410 unique, non-overlapping probes complementary to sense sequences only and a more inclusive *P*. *aeruginosa* probe-set comprising 88,641 unique probes complementary to both sense and the reverse complement of every other 100-mer sequence (Fig. 1B). Probe templates were chemically synthesized in parallel on a microarray and then cleaved from the array.

To confirm that there was no sequence bias in probe synthesis or amplification, we PCR amplified the pool of *M*. *tuberculosis* and *P*. *aeruginosa* probe templates. Sequence analysis of the amplified pools showed a narrow, even distribution across all mRNAs and ncRNAs for both *M*. *tuberculosis* and *P*. *aeruginosa* (Supplementary Fig. S1). We detected probes for 3,888 out of 3,906 (99.5%) annotated *M*. *tuberculosis* genes and 6 out of 20 annotated ncRNAs; the missing sequences could have been due to inefficient synthesis or inefficient PCR amplification of their corresponding probes. For *P*. *aeruginosa*, we detected probes for all mRNAs and ncRNAs. As expected, probes corresponding to rRNAs or tRNAs were absent from both sets.

For PatH-Cap enrichment, single-stranded, biotinylated RNA probes were made from the probe templates via *in vitro* transcription and hybridized in solution to standard dual RNA-seq libraries (pre-PatH-Cap). The excess of biotinylated RNA probes drives their hybridization to complementary targets^9^. The bacterial mRNA targets are then pulled down by their corresponding biotinylated RNA probe using streptavidin-coated beads, PCR amplified and sequenced (post-PatH-Cap). By sequencing pre-PatH-Cap libraries, dominated by host transcripts, and post-PatH-Cap libraries, enriched in bacterial mRNA transcripts, PatH-Cap enables analysis of both host and bacterial transcriptional profiles, respectively, from a single sample (Fig. 1A).

### PatH-Cap efficiently enriches for bacterial mRNA and increases confidence in the level of bacterial gene expression quantification

**Figure 2:**
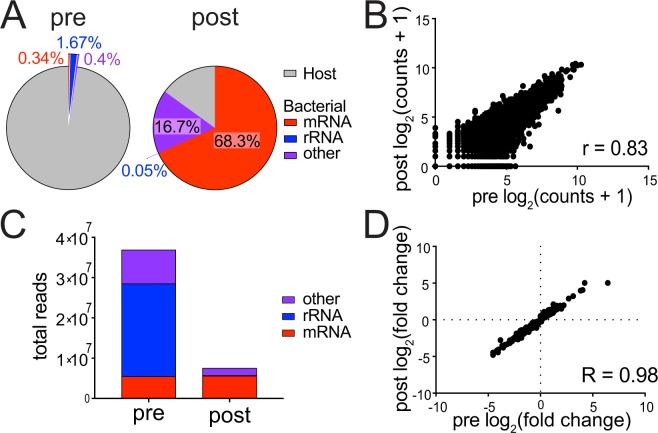
PatH-Cap enriches bacterial mRNA and depletes bacterial rRNA. (**A**) PatH-Cap enriches bacterial mRNA and depletes host and bacterial rRNA from the pre-PatH-Cap library to yield the post-PatH-Cap library. Other bacterial RNA includes ncRNA, tRNA and intergenic regions. (**B**) Gene expression correlation of bacterial log_2_(counts + 1) of pre- and post-PatH-Cap libraries. (**C**) Composition of pre- and post-PatH-Cap libraries from isoniazid treated (6 hr) *M*. *tuberculosis* RNA samples demonstrates efficient depletion of bacterial rRNA. (**D**) Differential expression analysis between untreated and antibiotic treated libraries from C pre- and post-PatH-Cap was performed with DESeq2. Spearman correlation (R) was calculated between the log_2_(fold change) expression under antibiotic treatment for genes that achieve a statistical cutoff post-PatH-Cap (p-adj < 0.01, 780 genes).

We assessed both the efficiency of PatH-Cap at enriching for bacterial mRNA from a dual RNA-seq library and its ability to preserve relative transcript abundance, thereby reflecting the starting library. We applied PatH-Cap to mock a library made from 1.25 ng total *M*. *tuberculosis* RNA spiked into 125 ng mouse RNA to simulate infection of ~12,500 host cells with 1 bacterium per cell (Supplementary Fig. S2). In the pre-PatH-Cap library, only 2.4% of 80 million total aligned reads mapped to the bacterial genome, with the majority corresponding to bacterial rRNA (70% of bacteria-aligned reads are bacterial rRNA; 1.67% of total aligned reads) and the minority to bacterial mRNA (14.4% of bacteria-aligned reads are bacterial mRNA; 0.34% of total aligned reads). In contrast, post-PatH-Cap, 85% of 40 million total aligned reads mapped to the bacterial genome, with the majority corresponding to bacterial mRNA (80% of all bacteria-aligned reads are bacterial mRNA; 68% of total aligned reads) (Fig. 2A). Thus, PatH-Cap resulted in a 35-fold enrichment in the percentage of reads that align to the bacterial genome and a 201-fold enrichment in the percentage of reads that align specifically to bacterial mRNA. Consistent with previous studies of solution hybridization selection^12^, we observed a higher frequency of PCR duplicates in the post-PatH-Cap library than in the pre-PatH-Cap library, which can skew transcript quantification. After removal of duplicate reads (see “Methods”), we observed a high correlation of unique bacterial gene counts between pre- and post-PatH-Cap libraries (r = 0.83; Fig. 2B), thus confirming that PatH-Cap preserves relative transcript abundance of the starting material. Furthermore, PatH-Cap is highly reproducible between enrichment of two replicate libraries and two independent enrichments of the same library (r > 0.98; Supplementary Fig. S3). Similar results were obtained for enrichment of the *P*. *aeruginosa* transcriptome using *P*. *aeruginosa*-specific probes (Supplementary Fig. S4) demonstrating the robustness of PatH-Cap across different pathogen transcriptomes and probe-sets.

To further examine the performance of PatH-Cap, we compared the number of sequencing reads required to observe (detect by ≥1 unique transcript) all bacterial genes present in pre- and post-PatH-Cap libraries by iteratively down-sampling bacteria-aligned reads (see “Methods”; Supplementary Fig. S2). The majority of bacterial genes, ~80% (~3,125), were observed at a lower sequencing depth post- as compared to pre-PatH-Cap (pre: 7.5 million reads; post: 0.2 million reads). Deep sequencing was insufficient to observe all 3,906 genes in either library (pre: 80 million reads observed 3,728 genes; post: 40 million reads observed 3,393 genes). The genes that were not observed post-PatH-Cap were the least abundant pre-PatH-Cap (Supplementary Fig. S2). To understand the basis for this loss, we performed PatH-Cap enrichment on a second mock library that contained 10-fold more bacterial mRNA compared to the first library (1.25 ng purified *M*. *tuberculosis* mRNA spiked into 125 ng total mouse RNA) (Supplementary Fig. S5). In this case, only 49 genes were not observed after PatH-Cap, which again corresponded to the least abundant genes in the pre-PatH-Cap library (Supplementary Fig. S5). The loss of genes after PatH-Cap enrichment is, thus, due to their extremely low abundance rather than the nature of the capture probes.

Although PatH-Cap slightly reduced the total number of observed genes, importantly, it increased the number of unique transcripts detected per gene (Supplementary Fig. S5). A higher number of unique transcripts per gene increases the certainty in gene expression measurement and, as a result, is a more reliable quantitation of transcript abundance because of the increase in the signal to noise ratio. This is particularly important in the analysis of low-input samples where technical variations can introduce noise that masks true biological differences^14^. Detection of ≥10 unique transcripts per gene increases the certainty and reliability in gene expression measurements^15^. In our mock library, at a sequencing depth of 3 million reads, the number of genes with ≥10 unique transcripts increased from 1,859 (48% of total genes) in the pre-PatH-Cap library to 3,305 (85% of total genes) in the post-PatH-Cap library demonstrating that PatH-Cap increases the level of detection and, therefore, provides a more reliable quantitation of transcript abundance (Supplementary Fig. S5).

Lastly, to confirm the ability of PatH-Cap to preserve differences in gene expression between two conditions, we applied PatH-Cap to RNA-seq libraries made from axenic antibiotic-treated bacterial cultures and untreated controls (Fig. 2C,D; Supplementary Fig. S4). DEseq2 differential expression analysis^16^ showed high correlation between the magnitude of expression changes of pre- and post-PatH-Cap samples (Spearman R = 0.98 for both; 740 *M*. *tuberculosis* genes, Fig. 2D; 633 *P*. *aeruginosa* genes, Supplementary Fig. S4). Importantly, this result demonstrates that differential expression analysis of post-PatH-Cap libraries reproduces analysis of pre-PatH-Cap libraries.

### PatH-Cap enables low-input and single eukaryotic cell dual RNA-seq studies

**Figure 3:**
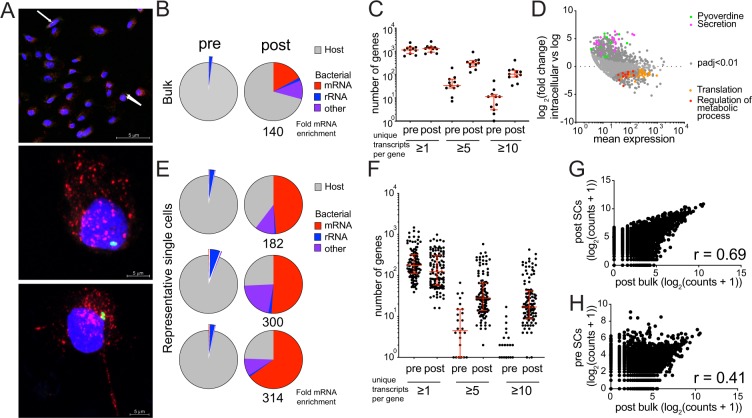
PatH-Cap enables analysis of low-input and single eukaryotic cell dual RNA-seq libraries. Epithelial cells were infected with *P*. *aeruginosa* PAO1-GFP at MOI = 25 for 1 hr followed by the addition of gentamicin for 1 hr. (**A**) Infected cells were stained with DAPI and anti-Lamp-1 antibodies to determine intracellular bacterial load (white arrows, top image). Few bacteria, 1–3, are observed per infected cell; two representative cells are shown with one and two bacteria (middle and bottom images, respectively). (**B**–**H**) GFP positive infected cells were FACS sorted as bulk populations (1,000 cells) or single cells. Dual RNA-seq libraries were made and enriched using *P*. *aeruginosa* specific probes. (**B**) Composition of pre- and post-PatH-Cap libraries for the average of all bulk populations. Fold enrichment of percentage of reads that align specifically to *P*. *aeruginosa* mRNA is shown. (**C**) Total number of *P*. *aeruginosa* genes detected with ≥1, ≥5 and ≥10 unique transcripts in pre- and post-PatH-Cap bulk libraries. Each dot represents a replicate. Bar shows median and error bars show interquartile range. (**D**) Gene expression of intracellular *P*. *aeruginosa* was compared to axenic cultures harvested at mid-logarithmic phase using DESeq2. GO term enrichment analysis was performed using the PANTHER database. MA plot shows mean expression and log_2_(fold change) in expression between intracellular bacteria and axenic log culture. Differentially expressed genes (p-adj < 0.05, 659 genes, dark gray) and those encoding genes belonging to pyoverdine biosynthesis (9, green), secretion (21, pink), regulation of metabolic processes (11, red) and translation (27, orange) GO terms are highlighted. (**E**) Composition of pre- and post-PatH-Cap libraries for three representative single eukaryotic cells as in (**B**). (**F**) Total number of *P*. *aeruginosa* genes detected with ≥1, ≥5 and ≥10 unique transcripts in pre- and post-PatH-Cap single-cell libraries as in (**C**). (**G**,**H**) Bacterial gene expression correlation of the sum of bulk library and the sum of all single cells post-PatH-Cap (**G**) and pre-PatH-Cap (**H**).

Having shown that PatH-Cap can efficiently enrich for bacterial mRNA and provides a reliable quantitation of transcript abundance, we sought to demonstrate its ability to enable analysis of low-input samples. Without PatH-Cap, such studies would not be possible because of the inability to obtain sufficient input material necessary for rRNA depletion and to reliably quantify the low fraction of bacterial transcripts. One such example is the study of intracellular *P*. *aeruginosa*, an opportunistic pathogen with the unique ability to cause both acute and chronic infections at mucosal surfaces. Although it is primarily considered an extracellular pathogen, it has been demonstrated that *P*. *aeruginosa* can invade and survive inside epithelial cells^17,18^, serving as a potential antibiotic-tolerant reservoir^19^. In a model of bladder epithelial cells infected with fluorescently labelled *P*. *aeruginosa* (PAO1-GFP), only ~5–10% of cells are infected with 1–3 bacteria each (Fig. 3A and Table S1), thus limiting the number of infected cells that can be isolated for input into library construction. We generated dual RNA-seq libraries from 1,000 GFP positive, FACS-sorted eukaryotic cells (3 biological replicates) and performed PatH-Cap using *P*. *aeruginosa* specific probes. This amount of RNA input is ~10-fold lower than our previously characterized mock samples.

Application of PatH-Cap to these libraries resulted in 140-fold enrichment of the percentage of reads that aligned to *P*. *aeruginosa* mRNA while maintaining relative transcript abundance (Fig. 3B and Supplementary Fig. S6). Of note, only 26% of host genes are observed in post-PatH-Cap libraries compared to 91% in pre-PatH-Cap libraries and the correlation between the host transcriptional program measured pre- and post-PatH-Cap is low (r = 0.59, data not shown), reinforcing the need to sequence both the pre-PatH-Cap library to characterize the host and the post-PatH-Cap library to characterize the bacteria. At the same sequencing depth, we observed a similar number of bacterial genes pre- and post-PatH-Cap (pre-PatH-Cap median: 1126; post-PatH-Cap median: 1293; Fig. 3C); however, the median number of genes detected with ≥10 unique transcripts increased from 11 in pre-PatH-Cap libraries to 103 in post-PatH-Cap libraries (genes with ≥5 unique transcripts increased from 33 to 310; Fig. 3C). Thus, for these low-input samples (1,000 host cells), we achieved a more reliable quantitation of transcript abundance after PatH-Cap even at a low sequencing depth of 2.5 million reads (Supplementary Dataset S1).

We compared the intracellular bacterial expression profiles from 1,000 infected cells after PatH-Cap to that of axenic *P*. *aeruginosa* cultures harvested at mid-logarithmic phase (Fig. 3D). GO (gene ontology) term enrichment analysis (FDR < 0.02)^20^ showed intracellular bacterial upregulation of genes involved in the biosynthesis of the siderophore pyoverdine, required for iron acquisition, as well as the type III secretion system, used to translocate effectors across the eukaryotic cell membrane. In contrast, genes involved in translation and regulation of metabolic processes were downregulated compared to growing bacteria, demonstrating that adaptations, likely in response to changes in nutrient availability, occur very early in infection. These results are in agreement with previous large-scale transcriptional analyses of *P*. *aeruginosa ex vivo* and *in vivo*^3,21,22^, thus validating PatH-Cap as a method to reliably profile bacterial gene expression from as few as 1,000 infected cells at low sequencing depth.

Recently, there has been significant interest in single eukaryotic cell expression analysis because of its ability to reveal heterogeneity during encounters between bacteria and host cells^6^. However, the only study that has performed paired, simultaneous host-pathogen profiling at the single-cell level required >10 infecting bacteria per cell, as well as analyses that assessed bacterial gene expression at the level of regulons to compensate for the low number of bacterial transcripts detected^8^. To ascertain PatH-Cap’s ability to enable bacterial profiling from single eukaryotic cells infected with 1–3 bacteria, we made libraries from individual, FACS-sorted *P*. *aeruginosa* infected epithelial cells (SCs) and compared data from PatH-Cap enriched SCs (Supplementary Dataset S2) and 1,000-cell bulk libraries. We confirmed the quality of the datasets obtained for the 115 SCs that passed our quality control filters (see “Methods”) by verifying that the correlation of host gene expression between the bulk libraries and the sum of the SC libraries before PatH-Cap enrichment was high (r = 0.80, Supplementary Fig. S6). In the pre-PatH-Cap libraries, the number of bacterial genes observed per infected cell (median 175 genes at median sequencing depth of 1.5 million reads per cell) was consistent with the previous report using the same library construction protocol on a mock sample approximating a single bacterium in a single eukaryotic cell (~300 genes)^8^. PatH-Cap enriched bacterial mRNA transcripts in these SC libraries as efficiently as in bulk libraries (222-fold and 140-fold, respectively, Fig. 3E and Supplementary Fig. S6). Although, we observed fewer genes post- than pre-PatH-Cap at the same sequencing depth (median: 123 genes; Fig. 3F), the number of bacterial genes detected with ≥5 or ≥10 unique transcripts per gene increased post-PatH-Cap (pre-PatH-Cap median: 4.5 genes ≥5, 0 genes ≥10; post-PatH-Cap median: 28 genes ≥5, 17 genes ≥10; Fig. 3F). The increase in the quantitation of transcript abundance resulted in a higher correlation between the post-PatH-Cap SC data and bulk data as compared to the pre-PatH-Cap SC data and bulk data (r = 0.69 vs 0.41, respectively; Fig. 3G,H).

We sequenced these libraries deeper and focused our analysis on 23 eukaryotic cells with >5,500 unique bacterial mRNA transcripts (~1X coverage). Expression of a common set of 26 genes was shared by all 23 single cells (Supplementary Table S2). These 26 genes are within the top 4% of the most highly expressed genes in the bulk samples and the majority (16 genes) are core essential genes^23^ encoding proteins involved in translation. While expanding studies to many more cells will be necessary to obtain statistically significant data to describe individual cell behavior beyond the most abundant mRNAs that may be present in every bacterial cell, we have, nevertheless, demonstrated that PatH-Cap improves the quantitation of bacterial transcript abundance in single eukaryotic cells, enabling its future application to studies of host-pathogen gene expression during infection at the single-cell level.

### PatH-Cap enables temporal analysis of paired host and bacterial transcriptional programs

Leveraging PatH-Cap’s ability to quantify bacterial mRNAs in infected host cells and to provide simultaneous characterization of the host from the same sample, we utilized PatH-Cap to transcriptionally profile host and bacterial transcriptomes throughout a time course of infection. We used a model of *M*. *tuberculosis* infection of murine bone marrow-derived macrophages (BMDMs) at a low multiplicity of infection (MOI). Key to *M*. *tuberculosis*’s pathogenic success is its ability to establish a replicative niche and grow within host macrophages. Previously, high MOI infection models (MOI ~10) were used to obtain sufficient bacterial RNA for transcriptional analysis of *M*. *tuberculosis* within the host^2,24–26^; however, these models result in rapid host cell death through distinct mechanisms^27,28^, precluding extended temporal profiling of infection and limiting previous analyses to comparisons of bacterial gene expression at early time points within the host to artificial *in vitro* growth conditions. By contrast, the use of a lower MOI (~1) results in greater host cell survival, establishment of intracellular bacterial niches that more closely resemble *in vivo* infection^29^, and allows for our temporal analysis of infection. We infected ~5 × 10^5^ BMDMs with *M*. *tuberculosis* (H37Rv-GFP) at MOI 1 and generated dual RNA-seq libraries from 6 replicates across six time points (4 hours post-infection and every 24 hours thereafter for up to 5 days post-infection), and sequenced the 36 libraries to a depth of at least 25 million reads pre-PatH-Cap and a depth of at least 4 million reads post-PatH-Cap (Supplementary Dataset S3).

**Figure 4:**
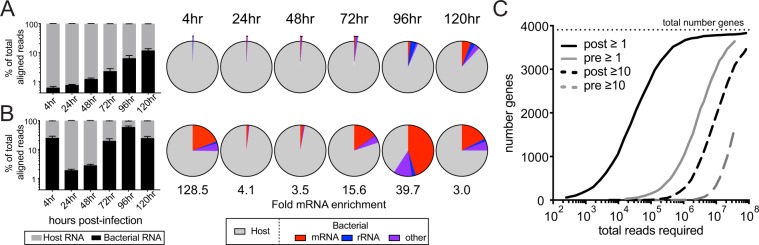
PatH-Cap enables temporal analysis of *M*. *tuberculosis* infection. BMDMs were infected with *M*. *tuberculosis* (H37Rv) at MOI 1. Cells were harvested 4 hours after *M*. *tuberculosis* addition to macrophages and every 24 hours thereafter for up to 5 days post-infection. Dual RNA-seq libraries were made and enriched using *M*. *tuberculosis* specific probes. (**A**,**B**) Average host and bacterial composition of pre-PatH-Cap libraries (**A**) and post-PatH-Cap (**B**) libraries at each time point (6 replicates per time point). Fold enrichment for percentage of reads that align specifically to *M*. *tuberculosis* mRNA is shown below. (**C**) Iterative down-sampling of bacteria-aligned reads of one representative replicate harvested at 4 hours shows the number of genes observed (solid lines) and those detected with ≥10 unique transcripts (dashed lines) at various sequencing depths for pre- (gray lines) and post-PatH-Cap (black lines) libraries.

The bacteria-derived fraction in each pre-PatH-Cap library increased over time from 0.7% of total aligned reads immediately after phagocytosis to 10% at 5 days post-infection, consistent with intracellular bacterial growth (Fig. 4A). PatH-Cap increased the percentage of bacterial aligned reads for each library, yielding a median 9.4-fold enrichment in reads that aligned specifically to bacterial mRNA (range 2.8–151-fold enrichment for all 36 libraries) and resulting in a considerable decrease in the sequencing cost for this experiment (Fig. 4A,B and Supplementary Table S3). Bacterial gene expression correlations between pre- and post-PatH-Cap libraries for each replicate were high (r = 0.77–0.95). Deep sequencing (~40 million reads) of the 4 hour pre-PatH-Cap libraries was insufficient to reliably quantify all bacterial genes, despite host and bacterial rRNA depletion using RiboZero during library construction: only 42% (1,650) of genes were quantified by ≥10 unique sequencing reads. In contrast, this same sequencing depth resulted in reliable quantification of 79% (3,097 with ≥10 unique sequencing reads) of bacterial genes in post-PatH-Cap libraries (Fig. 4C). We observed a median of 98% (3,840 genes) of *M*. *tuberculosis* genes across all 36 samples. Due to the low reliability in gene expression quantification, particularly at the 4 hour time point in pre-PatH-Cap libraries, we performed all subsequent analysis of bacterial transcriptional responses on post-PatH-Cap data.

**Figure 5:**
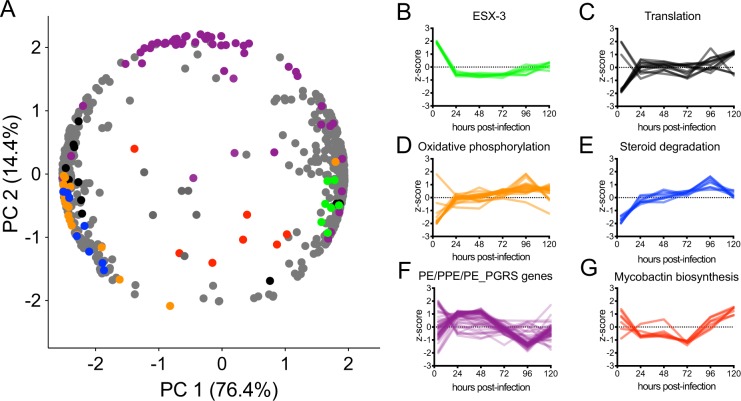
Transcriptional dynamics of intracellular *M*. *tuberculosis* growth. (**A**) PCA analysis of *M*. *tuberculosis* temporal gene expression for the 723 genes that showed at least a two-fold expression change throughout the time course and were detected above a minimal expression threshold (≥32 counts). Each gene’s coordinate position reflects its temporal expression pattern. Genes belonging to KEGG pathways shown in (**B**–**G**) are highlighted. Normalized temporal expression (z-score) of *M*. *tuberculosis* genes belonging to (**B**). ESX-3 secretion system (7, green), (**C**) Translation (13, black), (**D**) Oxidative phosphorylation (20, orange), (**E**) Steroid degradation (8, blue), (**F**) Antigen encoding PE/PPE/PE_PGRS gene family (52, purple) and (**G**) Mycobactin biosynthesis (7, red).

We first analyzed bacterial gene expression across all time points in the post-PatH-Cap data. We restricted our analysis to the 723 bacterial genes that showed at least a two-fold expression change between any two time points and were detected above a minimal expression threshold in at least one time point (see “Methods”). We then performed PCA analysis on each gene’s temporal expression pattern for these 723 genes, such that each gene’s coordinate position represents its expression kinetics (Fig. 5A). Genes with similar temporal behavior throughout infection appear close to one another in the PCA plot. The greatest source of variance, PC1 (76.4%), segregated genes based on their temporal behavior early in infection, specifically whether expression increased or decreased between the first two time points (4 and 24 hours post-infection), suggesting that the majority of *M*. *tuberculosis* transcriptional reprogramming occurs within 24 hours of phagocytosis, consistent with previous findings^25^. Genes with positive PC1 scores, such as the operon encoding the virulence related secretion system ESX-3, were repressed early in infection, while genes with negative PC1 scores were induced early in infection (Fig. 5B–E). PC2 (14.4% of variance) segregated genes based on other temporal behaviors. Genes with positive PC2 scores, corresponding to the bacterial antigen-encoding gene family of PE/PPE/PE_GRS, showed an early increase and then decreased throughout our time-course (Fig. 5F).

We hypothesized that functionally related genes might show similar temporal behavior. Indeed, we found that components of the ribosome (“Translation”) and aerobic cellular respiration (“Oxidative phosphorylation”) showed coordinated expression patterns, despite being organized in multiple, different operons (Fig. 5C,D). The expression of genes involved in both of these functions increased throughout infection, particularly between the 4 and 24 hour time points. The increase in expression of these growth-related processes is consistent with *M*. *tuberculosis* proliferation inside host macrophages. We then looked for other biological pathways that clustered with translation and oxidative phosphorylation to identify additional potential functions that might correlate with bacterial growth. Genes encoding proteins involved in steroid degradation showed a similar induction early in infection and a higher, more uniform increase in expression late in infection (Fig. 5E). This observation is consistent with a previous report that, upon infection of monocyte-like THP-1 cells, *M*. *tuberculosis* upregulates cholesterol catabolism. However, in contrast to the study that reported a concomitant upregulation of host genes involved in cholesterol biosynthesis with MOI 10^26^, we did not observe host induction of genes in this pathway in our pre-PatH-Cap libraries (Supplementary Fig. S7). This difference in host cell response may be attributable to the use of primary cells or lower MOI in our infections (BMDMs vs THP-1s; MOI 1 vs 10), resulting in lower intracellular bacterial burden and less depletion of host cholesterol compared to higher MOI infections.

**Figure 6:**
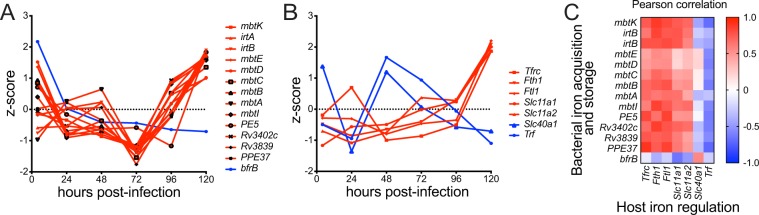
Expression of genes encoding bacterial iron-acquisition and host iron-regulation proteins are correlated. (**A**) Normalized temporal expression (z-score) of *M*. *tuberculosis* genes encoding proteins involved in iron acquisition (*mbtK*, *irtA*, *irtB*, *mgtE*, *mgtD*, *mgtC*, *mgtB*, *mbtA*, *mbtI*, *PE5*, *Rv3402C*, *Rv3839* and *PPE37* shown in red) and iron storage (*bfrB* shown in blue). (**B**) Normalized temporal expression of host genes encoding proteins involved in intracellular iron regulation, storage and export of iron from phagosomal and endosomal compartments (*Tfrc*, *Fth1*, *Ftl1*, *Slc11a1* and *Slc11a2* shown in red) and in extracellular iron export and import (*Slc40a1* and *Trf* shown in blue). (**A**) Heatmap showing Pearson correlation of temporal expression patterns of bacterial genes encoding proteins involved in iron acquisition and storage (shown in **A**) and host genes encoding proteins involved in iron regulation (shown in **B**).

In addition to carbon utilization, *M*. *tuberculosis* must acquire other essential nutrients, such as iron, to grow in the macrophage^30^. Consistent with previous reports that intracellular bacterial upregulate iron siderophore biosynthesis relative to *in vitro* logarithmically growing bacteria^2^, we found that genes involved in bacterial mycobactin biosynthesis, an *M*. *tuberculosis* iron siderophore, were highly expressed early after phagocytosis by macrophages (Fig. 5G). However, we also found that expression of genes involved in iron acquisition and transport increased even more dramatically later in infection (96 and 120 hours post-infection; Fig. 6A), a phenomenon that has not been previously described because of the inability to transcriptionally profile *M*. *tuberculosis* late in infection. In contrast, *bfrB*, which encodes the bacterial iron storage protein bacterioferritin that is required for survival under *in vitro* iron starvation^30^, decreased in expression during the course of infection. When we examined the paired host transcriptional response in our paired pre-PatH-Cap libraries, we found that host genes encoding proteins involved in iron regulation in infected, but not uninfected macrophages, were highly correlated with the temporal expression pattern of *M*. *tuberculosis* genes involved in iron acquisition (Fig. 6B,C; Supplementary Fig. S8). Infected macrophages showed late induction of *Slc11a1*, *Slc11a2* and *Tfrc* encoding the transporters Nramp1, Nramp2 and transferrin receptor, which regulate cytosolic accumulation of iron^31^ (Supplementary Fig. S8). Cytosolic iron may be bound by *Ftl1* and *Fth1* encoded ferritin, which are also induced late in infection. Together, upregulation of genes encoding Nramp1, Nramp2, transferrin receptor and ferritin suggests sequestration of intracellular iron in the macrophage cytoplasm. Meanwhile, downregulation of *Slc40a1* and *Trf*, encoding the extracellular iron exporters ferroportin and transferrin respectively, could act in concert to slow iron flux into *M*. *tuberculosis*-infected macrophages resulting in sequestration of iron away from phagosomal localized *M*. *tuberculosis*.

Taken together, this paired analysis describes a host-pathogen dynamic interplay in which macrophages, upon sensing intracellular *M*. *tuberculosis* infection, attempt to restrict bacterial growth by limiting iron availability in the phagosome late in infection while, at the same time, *M*. *tuberculosis* induces iron scavenging functions. Indeed, a *M*. *tuberculosis* mycobactin biosynthesis mutant, unable to scavenge free extracellular iron, has been reported to be attenuated for growth in THP-1 cells late in infection^32^, demonstrating the importance of the battle for iron between host and pathogen, particularly late in infection. Our paired transcriptional profiling of intracellularly replicating *M*. *tuberculosis* and the corresponding host response to infection represents the first integrated, temporal analysis of *M*. *tuberculosis* and host transcriptomes from the same sample and the data will serve as a resource for future studies.

## Discussion

Examining the paired transcriptional responses of host and pathogen provides insight into their dynamic interaction by unveiling molecular pathways that are at play during these encounters. However, due to the scarcity of bacterial mRNA within infected cells, accurate profiling of bacterial transcriptional programs in the context of infection has been technically challenging. We have developed Pathogen Hybrid Capture (PatH-Cap) to enrich for bacterial mRNA from dual RNA-seq libraries. By enriching bacterial mRNA and depleting bacterial rRNA in a single step, we obviate the need for a separate rRNA depletion step as had been previously required^7^, facilitating the study of low-input and low-pathogen burden samples. Sequencing of pre-PatH-Cap libraries, to obtain the host transcriptional program, and post-PatH-Cap libraries, to obtain the bacterial transcriptional program, enables paired characterization of host and pathogen from the same sample. Although the use of PatH-Cap requires sequencing of two separate libraries, neither one needs to be sequenced exceedingly deep to enable comparative gene expression analysis^33^, as they are comprised primarily of the desired material resulting in decreased sequencing costs. We show that PatH-Cap is reproducible, efficient at enriching for bacterial mRNA, robust across different bacterial species, and able to recapitulate bacterial gene expression profiles from the original sample. Importantly, PatH-Cap increases the number of unique transcripts per gene providing a more reliable quantitation of transcript abundance at lower sequencing depth. It, thus, enables dual RNA-seq studies of host-pathogen interactions that were previously limited by the extremely deep sequencing required to reliably profile the pathogen.

One of the requirements for PatH-Cap to be successful is a starting library that accurately represents the complexity of the original biological sample. The efficiency of PatH-Cap is dependent on target abundance in the pre-PatH-Cap library, which is itself dependent on transcript capture and amplification during library construction. Exponential PCR amplification during library construction can reduce the abundance of rare transcripts to an even lower fractional abundance^34^, which can further decrease the efficiency of their capture by PatH-Cap. Therefore, limiting the number of PCR cycles during library construction is key to successful PatH-Cap enrichment. Consistent with previous studies of solution hybridization selection, analysis of post-PatH-Cap libraries should take into account PCR duplicate reads either through computational collapse^35^, as we did for our mock libraries and temporal analysis of *M*. *tuberculosis* infection, or the use of UMIs^36^, as we did for our analysis of *P*. *aeruginosa* infection. Future improvements in library construction protocols, particularly in transcript capture efficiency, will help address the low fractional abundance of bacteria-derived reads in dual RNA-seq libraries.

Development of new methods to analyze host and bacterial gene expression datasets remains a challenge for dual RNA-seq studies. In single eukaryotic cells infected by one or a few bacteria, variability in the measurements of unique sequencing reads corresponding to true heterogeneous transcript abundance per cell is difficult to distinguish from noise. This is exacerbated by the fact that a low number of read counts traditionally demonstrate greater dispersion and are, therefore, inherently noisy^16^. A single bacterium is estimated to contain 10^3^–10^5^ total mRNA molecules with 0.1 to 1 mRNA transcripts per gene^37^, implying that the true number of mRNA molecules for the majority of bacterial genes is low or even zero. Thus, we are currently limited to the analysis of highly expressed bacterial genes, such as those that are reliably quantified after PatH-Cap. Higher throughput library construction methods are needed to dramatically increase the number of cells that can be profiled to achieve increased statistical power for expression analysis. Additionally, novel computational approaches are required to deal with the sparse data obtained from a single bacterium, such as imposing structures in estimated gene expression through genetic pathway-based or manifold regularization^38^. Finally, tools for integrated analysis of host and pathogen transcriptional profiles are needed to facilitate thorough, paired analysis of host-pathogen interactions. Efforts thus far, including our study of macrophage and *M*. *tuberculosis* transcriptional responses, have been hypothesis-driven^8,39^. Novel, unbiased integration of host and pathogen transcriptional responses will be required to transform our understanding of the heterogeneity in these complex interactions.

Hybridization capture as a general strategy has been a powerful method to enrich for lowly abundant targets of interest^9–11^. Recent applications in infection include enrichment for total bacterial RNA (Path-seq)^7^ and viral genomic fragments (CATCH)^12^ from mixed host-pathogen samples. Notably, because our strategy excludes rRNA and tRNA capture, prior depletion of rRNA is not necessary, enabling analysis of low-input samples, including single eukaryotic cells. Path-seq, by comparison, requires pooling of 30 mice per replicate to obtain sufficient material (300 μg) for the necessary rRNA depletion step in contrast to the ~10 pg requirement of PatH-Cap. Nevertheless, a caveat of both approaches is that a reference genome is required as probe design relies on annotated transcripts; unannotated genes would not be enriched. Additional applications of this strategy include exclusive enrichment and characterization of a targeted subset of transcripts such as bacterial ncRNAs, which constitute an even smaller fraction of total RNA and have been shown to play an important role in host-pathogen interactions^40^. More broadly, this enrichment strategy can be adapted to transcriptionally profile specific species in mixed microbial communities, such as the microbiome, where post-hoc sequence alignment will provide an additional layer of specificity. We propose that by varying probe design to target specific nucleic acids and taking into account factors that can contribute to loss of rare transcripts, this strategy can be applied to capture an even greater range of transcripts in complex, mixed samples.

## Materials and Methods

### Probe design and synthesis

For *M*. *tuberculosis*, coding DNA sequences (CDS) and annotated ncRNA sequences from the RefSeq annotation NC_000962 were used. For each region, 100 bp sequences were tiled end to end with no overlap. In cases where the length of the transcript was not divisible by 100, the remaining uncovered area was evenly distributed at the 5′ and 3′ end. One CDS transcript was excluded because it was shorter than 100 bp. A total of 38,410 probes were designed. For *P*. *aeruginosa*, using the RefSeq annotation NC_002516, all sequences annotated as “5 S”, “16 S” or “23 S” rRNA or “tRNA” were removed. Then, sequences annotated as CDS and ncRNAs were elongated evenly from the 3′ and 5′ end to generate multiples of 100 bp. Hundred-mer probes were tiled across each region with no overlap; the reverse complement of every other probe was generated. Homologous probes were omitted resulting in 88,641 unique probes. Adapters were added at the end of each 100 mer to serve as PCR handles: 5′adapter = ATCGCACCAGCGTGT; 3′adapter = CACTGCGGCTCCTCA. The complex pool of 130-mer oligonucleotides was synthesized in parallel on a microarray by CustomArray, Inc. To synthesize RNA baits, oligos were PCR amplified using a primer that added a T7 promoter (Forward primer: GGATTCTAATACGACTCACTATAGGGATCGCACCAGCGTGT; Reverse primer: TGAGGAGCCGCAGTG). *In vitro* transcription in the presence of biotin-UTP was then used to generate biotinylated single-stranded RNA molecules which were aliquoted and frozen at −80 °C.

### RNA isolation

Bacteria were grown to mid-logarithmic phase, harvested by centrifugation, resuspended in TRIzol (ThermoFisher Scientific), incubated for 10 minutes at room temperature, transferred to tubes with zirconia beads, and bead beat three times for 60 seconds each, with 60 seconds incubations on ice in between pulses. RNA was extracted using the Directzol RNA extraction kit (Zymo Research). RNA preparation from mammalian cells was performed as described for bacteria, but with the omission of bead beating.

### *P*. *aeruginosa* infections, FACS sorting and microscopy analysis

For *P*. *aeruginosa* infections, 5637 human bladder epithelial cells (RRID: CVCL_0126) were seeded in 6 well plates overnight in RPMI supplemented with 10% FBS. Log-phase *P*. *aeruginosa* PAO1-GFP (GFP expression driven by a constitutive insulated promoter^41^) grown in LB was used to infect at MOI 25. Cells were incubated for 1 hour, extracellular bacteria were removed and media containing 200 μg/ml gentamicin was added. For FACS sorting, cells were detached using trypsin, sorted into 96-well plates containing lysis buffer (RNA-Gem Lysis buffer, 1% BME and RNAse inhibitor) and frozen. For microscopy, cells were seeded on glass coverslips, infected in the same manner, fixed with 1% formaldehyde, stained with anti-LAMP-1 antibodies (RRID: AB_775978) and DAPI, imaged using a Zeiss LMS800 confocal microscope and acquired and analyzed using the Zeiss ZEN software. Total cells, infected cells and intracellular bacterial load were determined manually. Data represent two independent experiments.

### M. tuberculosis infections

*M*. *tuberculosis* strain H37Rv-GFP^42^ was cultured at 37 °C in Middlebrook 7H9 broth supplemented with 10% Middlebrook OADC, 0.2% glycerol, and 0.05% Tween-80. Murine bone marrow-derived macrophages (BMDMs) were prepared as previously described^43^ from C57BL6 mice (Jackson Laboratories) in accordance with protocol number 2007N000048 approved by Massachusetts General Hospital Institutional Animal Care and Use Committee. For infections, BMDMs were seeded in 6 well plates overnight in DMEM supplemented with 25 ng/ml rmM-CSF (R&D Systems). *M*. *tuberculosis* was grown to mid-log phase, washed once in PBS and resuspended thoroughly. A low-speed spin (500 rpm) was performed to pellet clumps. Bacteria were added to DMEM with 20% heat-inactivated horse serum to yield a multiplicity of infection of 1. After 4 hours, extracellular bacteria were removed with washes and fresh media was added. All *M*. *tuberculosis* infections were conducted using BL3 practices and containment equipment according to protocol IBC-2016-00095-1 approved by the Institutional Biosafety Committee of the Broad Institute.

### Dual RNA-seq sample isolation and library construction

For validation experiments, bacterial and mammalian RNA was mixed at reported ratios and libraries were generated using the RNAtag-Seq protocol^44^. For *P*. *aeruginosa* infection experiments, 12 bulk populations of 1,000 cells (four technical replicates of 1,000 cells from three independent biological infections) and 288 single cells (96 single cells from each of three independent biological infections) were sorted. Samples were lysed by incubating at 75 °C for 5 minutes. Dual RNA-seq libraries were made using the sc-Dual-seq protocol^8^. For *M*. *tuberculosis* time course experiment, samples were harvested 4 hours after *M*. *tuberculosis* addition to macrophages and every 24 hours thereafter for up to 5 days post-infection (6 time points total). Six replicates of infected cells and three replicates of uninfected cells were used for each time point. Lysis and RNA isolation were performed as described. Libraries were generated using the RNAtag-Seq protocol^44^. During library construction, host and bacterial rRNA was depleted using a 1:5 mix of bacterial and eukaryotic RiboZero beads per the manufacturer’s protocol.

### Solution hybridization selection

Our hybridization reaction protocol was adapted from Gnirke *et al*.^9^. For each reaction, 30–300 ng of dual host-pathogen RNA-seq libraries was incubated with 2.5 µg mouse Cot-1 DNA, 2.5 µg sonicated salmon sperm DNA, 400 µM blocking primers (sequences complementary to library adapters: RNAtag-Seq: Fwd: AATGATACGGCGACCACCGAG

ATCTACACTCTTTCCCTACACGACGCTCTTCCGATCT; Rvs: CAAGCAGAAGACGGC

ATACGAGATNNNNNNNNGTGACTGGAGTTCAGACGTGTGCTCTTCCGATCT; scDual-seq Fwd: AATGATACGGCGACCACCGAGATCTACACGTTCAGAGTTCTA

CAGTCCGA, Rvs: CAAGCAGAAGACGGCATACGAGATNNNNNNGTGACTGGAGTT

CCTTGGCACCCGAGAATTCCA) and 500 ng biotinylated RNA bait in hybridization buffer (1X SSPE, 1X Denhardt’s, 0.2% SDS, 10 mM EDTA). This mixture was incubated at 68 °C for 16–72 hours. Samples were then incubated with streptavidin Dynabeads (M-280). Library fragments not hybridized to the biotinylated RNA bait were removed as follows: one wash with 1X SSC with 0.1% SDS at RT, and three washes with 0.1X SSC with 0.1% SDS at 68 °C. Enriched templates were purified and PCR amplified using universal primers that maintained library barcodes.

### Sequencing, alignment and analysis

Paired-end sequencing of pre- and post-PatH-Cap libraries was performed on Illumina NovaSeq and NextSeq platforms at the Broad Institute Genomics Core. Reads were aligned to the H37Rv or PAO1 genome from Refseq (NC_000962 and NC_002156 respectively) using BWA^45^ and the mouse or human transcriptome generated from Ensembl gene annotations (GRCm38/mm10 and GRCh38/hg38 respectively) using BBMap^46^. For RNAtag-Seq libraries both read1 and read2 were aligned; for scDual-seq libraries only read2 was aligned after trimming reads with stretches of 7 or more A’s. An in-house script was used for enumeration and metrics generation. Aligned reads that were determined to be the result of PCR duplication during library construction were collapsed into a single read so as to be counted only once. For scDual-seq libraries, transcripts with the same Unique Molecular Identifier (UMI) were collapse into a single read using an in-house script by clustering with respect to UMI, strand and gene boundary for eukaryotes or UMI and 5-prime mapped genomic location for prokaryotes. Then clusters were collapsed into single UMI normalized transcripts. For RNAtag-Seq, we used the MarkDuplicates function from the Picard toolkit^47^ to aggregate reads with the same 5-prime location into a single read. During this process, fragments with only one mapped read were removed for improved accuracy. All gene expression correlation analysis (Pearson r calculation) was done after duplicate reads were removed.

For analysis of bulk and single-cell scDual-seq libraries, we filtered out libraries based on host alignment of pre-PatH-Cap libraries: percent protein coding regions <45%; percent sense protein coding regions >65%; unique host transcripts (UMI collapsed) in protein coding regions >3,000. Eleven (11) bulk populations and 115 single cells passed these quality filters.

Differential expression analysis was conducted with DESeq2^16^. For analysis of intracellular bulk *P*. *aeruginosa* libraries, one replicate per biological sample was used. Only genes observed  (>0 counts) in at least two of three replicates of intracellular bacteria were analyzed (1,522 genes).

### Generation of sequencing saturation curves

Reads aligned to bacterial transcriptome were randomly resampled at various read depths. Enumeration and computational collapse of duplicate reads was done at each read depth as described previously.

### Gene normalization and PCA analysis of *M. tuberculosis* intracellular gene expressio*n*

For temporal expression analysis of intracellular *M*. *tuberculosis*, the expression levels of all genes were normalized using rlog function (blind = FALSE) in the R DESeq2 package^16^, and replicates were averaged for each gene. Genes with a fluctuation likely to be dominated by random perturbations were removed. First, we excluded lowly expressed genes not detected above the minimal expression threshold of ≥32 counts in at least one time point post-PatH-Cap since they may suffer from an inflated variance at such low count level. Second, we kept only genes with an expression range higher than 1 (equivalent to a two-fold change in expression), so that genes with constant expression across time were removed. Among 3,906 genes in the post-PatH-Cap data, 2,910 genes passed the first filter, and, of these, 723 genes passed the second filter and were used for downstream analysis. For each gene, the expression values were then standardized with respect to the time course, to capture the dynamics rather than absolute levels. We then extracted principal components through the princomp function in the R stats package. Genes were represented by the first two principal components which captured 90.8% of variation. Designation of bacterial genes as “translation,” “oxidative phosphorylation” or “steroid degradation” was based on KEGG annotation. Designation of bacterial genes as “iron acquisition and storage” and host genes as “iron regulation” was based on literature reports^30,48^.

## Supplementary information

Supplementary Figures

Supplementary Dataset 1

Supplementary Dataset 2

Supplementary Dataset 3

## Data Availability

Sequencing datasets are available in the Sequence Read Archive (SRA): • Development of PatH-Cap: SRA accession PRJNA574614. • Dual transcriptional profiling of human bladder epithelial cell line (5637) infected with Pseudomonas aeruginosa (PAO1-GFP) sorted two hours post infection: SRA accession PRJNA574611. • Bone Marrow derived macrophages infected with Mycobacterium Tuberculosis: SRA accession PRJNA573678. Pipelines and scripts for alignment and analysis can be found at: • Alignment, counting and metrics generation: https://github.com/broadinstitute/PatHCap_PL • UMI normalization: https://github.com/broadinstitute/UMINormalize • BAM sampler: https://github.com/broadinstitute/bam_sampler • Fastq sampler: https://github.com/broadinstitute/fastq_sampler • Probe design: https://github.com/broadinstitute/PatHCapProbes.
