# Supplementary material for: Hybridization-based capture of pathogen mRNA enables paired host-pathogen transcriptional analysis
Source: Sci Rep. 2019 Dec 17;9:19244. doi: 10.1038/s41598-019-55633-6 (PMC6917760; doi:10.1038/s41598-019-55633-6)
Supplement: Supplementary file 1 — Supplementary Figures [file 41598_2019_55633_MOESM1_ESM.pdf]

Hybridization-based capture of pathogen mRNA enables paired host-pathogen transcriptional analysis

Viktoria Betin[#1,2], Cristina Penaranda[#1,3,4,*], Nirmalya Bandyopadhyay[1], Rui Yang[1], Angela Abitua[1], Roby P. Bhattacharyya[1,5], Amy Fan[1], Roi Avraham[1,6], Jonathan Livny[1], Noam Shoresh[1], Deborah T. Hung[1,3,4,*]

Supplementary Figures and Tables

Supplementary Figure S1

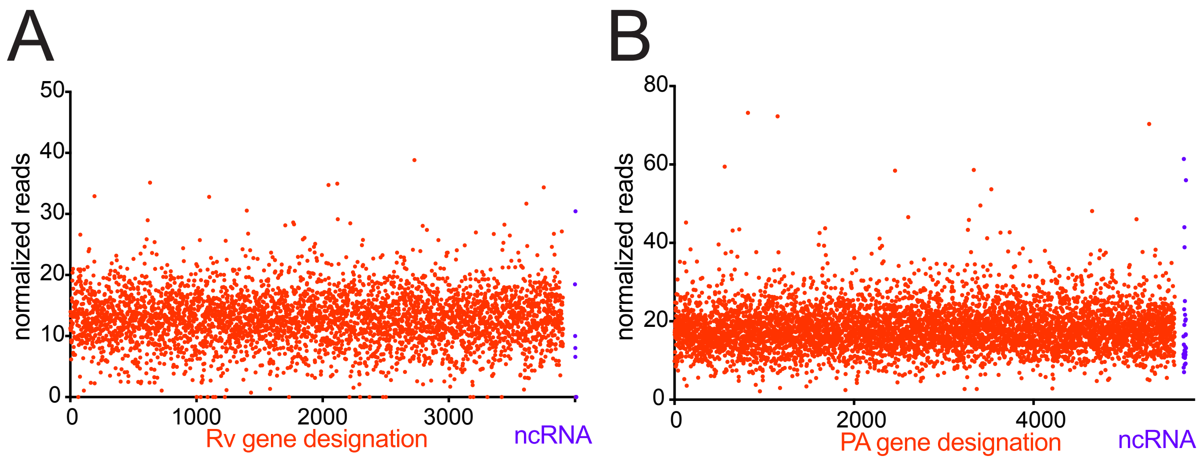

Supplementary Figure S2

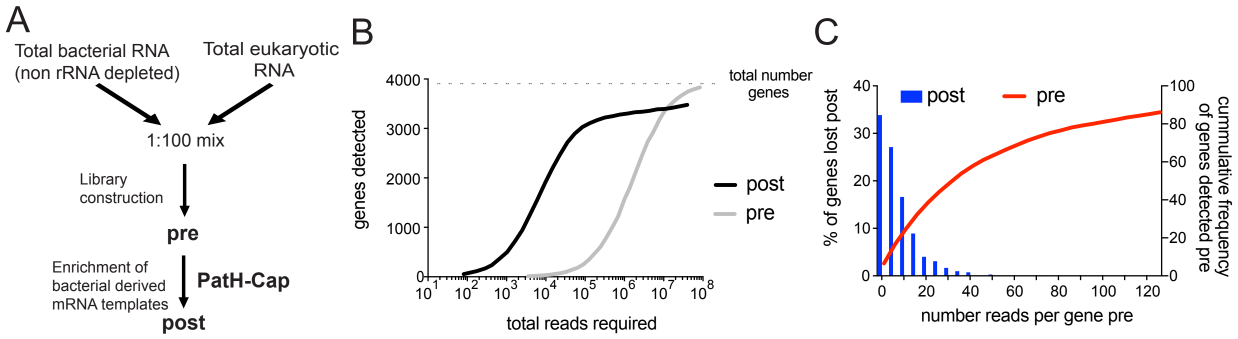

Supplementary Figure S3

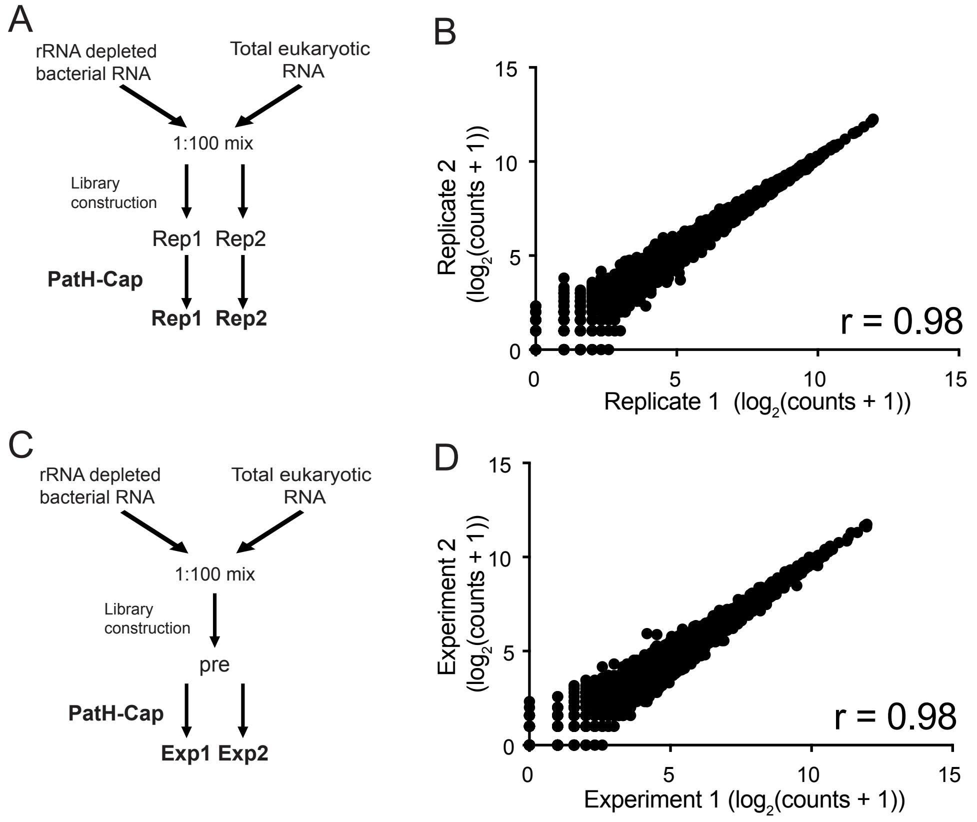

Supplementary Figure S4

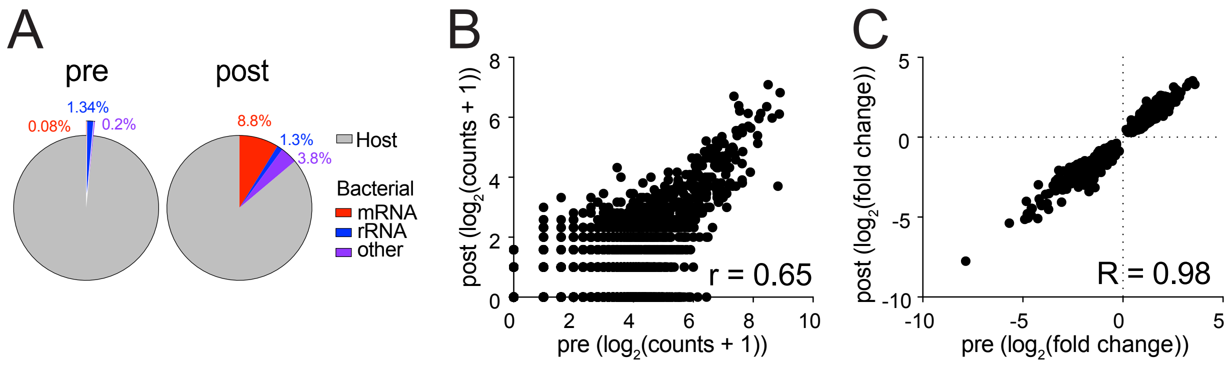

Supplementary Figure S5

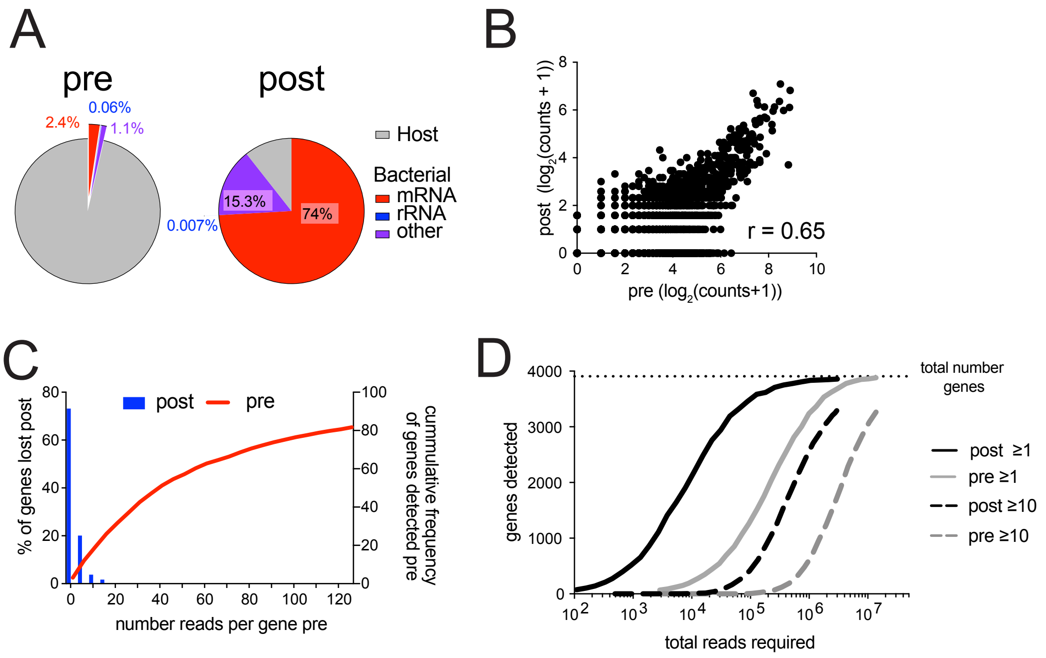

Supplementary Figure S6

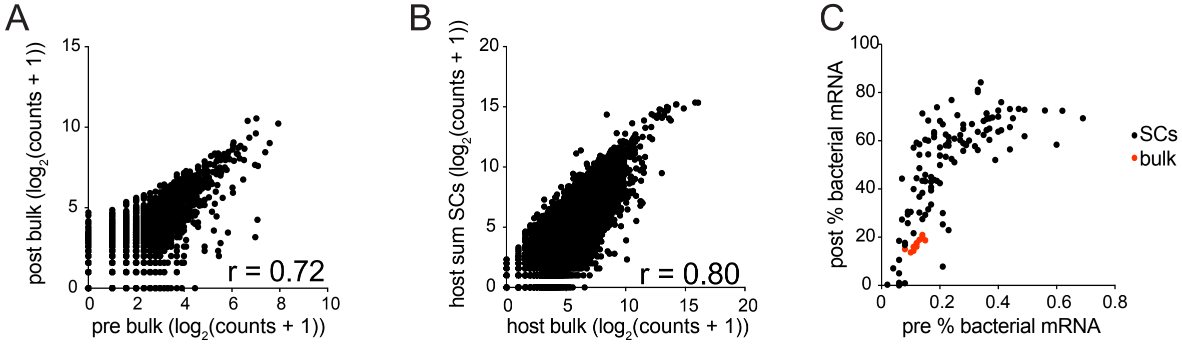

Supplementary Figure S7

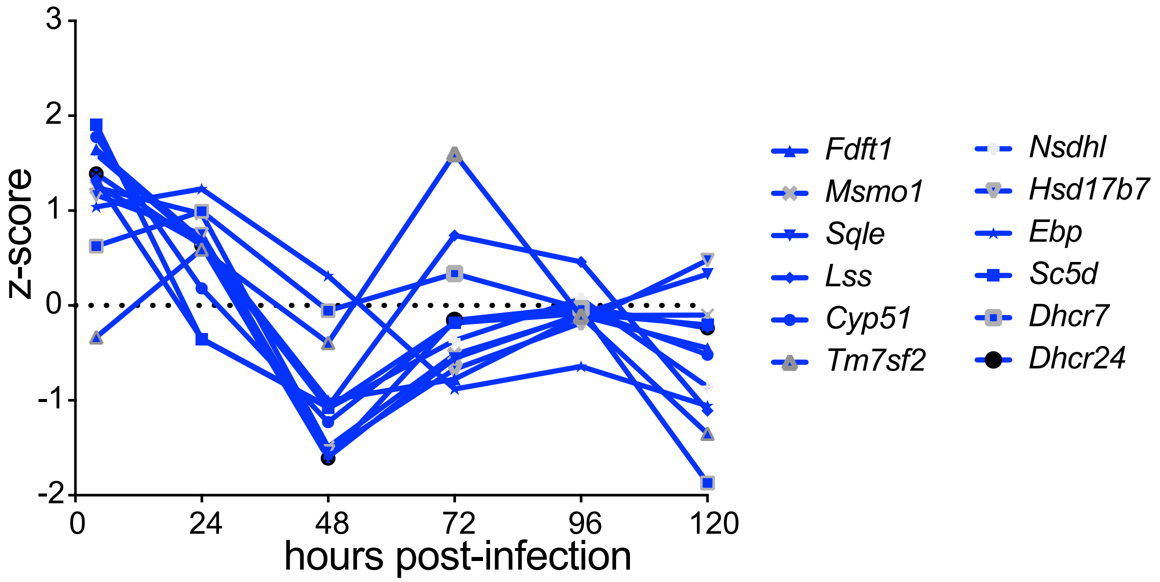

Supplementary Figure S8

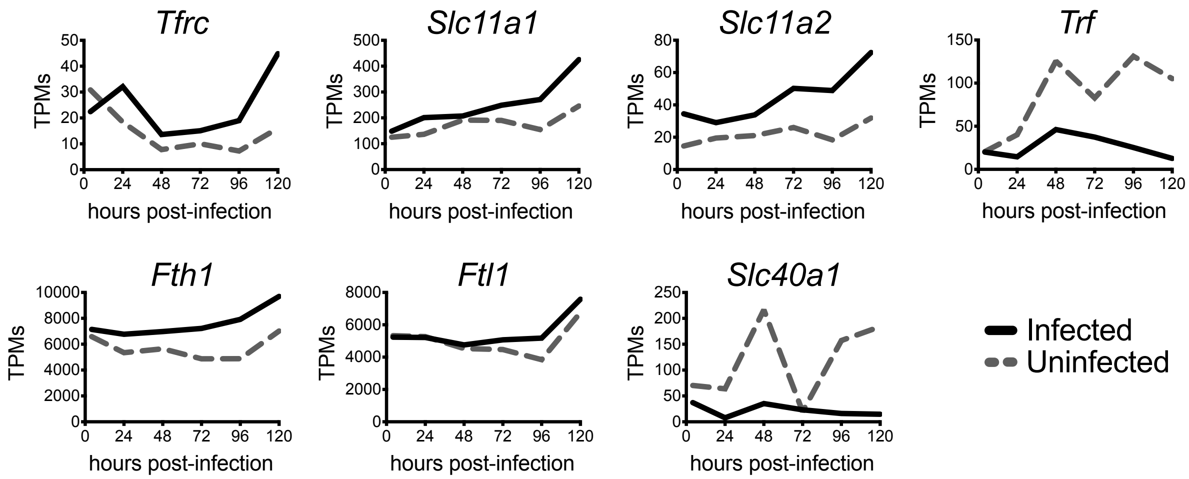

Supplementary Table S1

Sample	Uninfected	1 bacterium	2 bacteria	3 bacteria
1	246 *(89.5%)*	14 *(5.1%)*	14 *(5.1%)*	1 *(0.4%)*
2	499 *(94.3%)*	12 *(2.3%)*	15 *(2.8%)*	3 *(0.6%)*
3	470 *(94.6%)*	13 *(2.6%)*	12 *(2.4%)*	2 *(0.4%)*
Total *(Average)*	1215 *(92.8%)*	39 *(3.3%)*	41 *(3.4%)*	6 *(0.4%)*

Supplementary Table S2

PAO1 Locus Tag	Gene Name	Protein product description	Rank in bulk (out of 5,573)	Core Essential?*
PA4265	*tufA*	elongation factor Tu	1	ND
PA4277	*tufB*	elongation factor Tu	2	ND
PA1777	*oprF*	porin	3	no
PA4266	*fusA1*	elongation factor G	4	yes
PA2743	*infC*	translation initiation factor IF-3	5	yes
PA4257	*rpsC*	30S ribosomal protein S3	6	yes
PA4243	*secY*	preprotein translocase subunit	8	yes
PA4270	*rpoB*	DNA-directed RNA polymerase subunit beta	9	yes
PA4238	*rpoA*	DNA-directed RNA polymerase subunit alpha	10	yes
PA4239	*rpsD*	30S ribosomal protein S4	12	yes
PA4740	*pnp*	polynucleotide phosphorylase	13	yes
PA4262	*rplD*	50S ribosomal protein L4	17	yes
PA5239	*rho*	transcription termination factor Rho	18	yes
PA4267	*rpsG*	30S ribosomal protein S7	19	yes
PA1092	*fliC*	flagellin type B	20	no
PA4264	*rpsJ*	30S ribosomal protein S10	22	yes
PA4268	*rpsL*	30S ribosomal protein S12	25	yes
PA4269	*rpoC*	DNA-directed RNA polymerase subunit beta	29	yes
PA5015	*aceE*	pyruvate dehydrogenase subunit E1	32	no
PA4568	*rplU*	50S ribosomal protein L21	44	no
PA2494	*mexF*	resistance-nodulation-cell division 28RND29 multidrug efflux transporter	47	no
PA4274	*rplK*	50S ribosomal protein L11	53	yes
PA4273	*rplA*	50S ribosomal protein L1	72	yes
PA5291	*betT2*	choline transporter	96	no
PA0865	*hpd*	4-hydroxyphenylpyruvate dioxygenase	170	no
PA5362	-	phosphate regulon metal ion transporter containing CBS domains	191	no

Supplementary Table S3

	**Deep sequencing**	**PatH-Cap**
Median % bacterial mRNA	0.77%	16.2%
Reads per sample*	387,207,263	18,524,944
Total reads	13,939,461,474	666,897,977
Cost of sequencing**	$ 40,118	$ 1,919
Cost of PatH-Cap probes[#]	none	$ 5,000
**Total cost**	**$ 52,154**	**$ 6,919**

SUPPLEMENTARY FIGURE LEGENDS

Supplementary Figure S1. Designed probes provide even coverage of bacterial transcriptomes. a-b. Chemically synthesized transcriptome-specific probes for *M. tuberculosis* (A) and *P. aeruginosa* (B) were PCR amplified and sequenced. Reads were aligned to pathogen genomes and number of read counts was normalized to gene length and sequencing depth to calculate the normalized number of reads per gene or ncRNA.

Supplementary Figure S2. PatH-Cap enriches for bacterial mRNA-derived transcripts. A. A dual RNA-seq library was made from a mock sample containing 1.25 ng non-rRNA depleted *M. tuberculosis* total RNA spiked into 125 ng mouse RNA mimicking 12,500 cells infected at a host:bacterial ratio of 1:1, and enrichment using PatH-Cap with *M. tuberculosis* specific probes was performed. **B.** Iterative down-sampling of bacteria-aligned reads shows the number of genes observed at various sequencing depths for pre- and post-PatH-Cap libraries from Fig 2A. **C.** Number of reads (bins of 5) in pre-PatH-Cap library corresponding to the genes not observed in post-PatH-Cap library from Fig 2A shown in blue bars. Cumulative frequency of genes observed in the pre-PatH-Cap library shown in red line.

Supplementary Figure S3. PatH-Cap enrichment is highly reproducible. A. Two replicate dual RNA-seq libraries made from the same starting material (purified *M. tuberculosis* mRNA spiked into mouse RNA at 1:100 ratio) were enriched using *M. tuberculosis* specific probes. **B.** Gene expression correlation of replicate post-PatH-Cap libraries. **C.** One of the libraries from A was enriched in an independent experiment. **D.**

Gene expression correlation of post-PatH-Cap libraries from the two independent experiments.

Supplementary Figure S4. PatH-Cap using *P. aeruginosa*-specific probes similarly enriches for bacterial mRNA-derived transcripts. A. A dual RNA-seq library made from 1.25 ng non-rRNA depleted *P. aeruginosa* total RNA spiked into 125 ng mouse RNA was enriched using *P. aeruginosa* specific probes. Pie charts show composition of the library pre- and post-PatH-Cap. **B.** Gene expression correlation of pre- and post-PatH-Cap libraries from A. **C.** Triplicate RNA-seq libraries were made from *P. aeruginosa* untreated or treated with gentamycin for 30 min and enriched using *P. aeruginosa*-specific probes**.** Differential expression analysis between untreated and antibiotic treated libraries before and after PatH-Cap was performed with DESeq2. The correlation between the $\log_2$(fold-change) expression under antibiotic treatment for genes that achieve a statistical cutoff post-PatH-Cap (p-adj<0.0001, 663 genes) is shown.

Supplementary Figure S5. PatH-Cap efficiency depends on target abundance. A dual RNA-seq library made from purified *M. tuberculosis* mRNA spiked into mouse RNA at 1:100 ratio was enriched using *M. tuberculosis* specific probes. **A.** Pie charts show composition of the library pre- and post-PatH-Cap. Other bacterial RNA includes ncRNA, tRNA and intergenic regions. **B**. Gene expression correlation of bacterial $\log_2$(counts + 1) pre- and post-PatH-Cap. **C.** Number of reads (bins of 5) in pre-PatH-Cap library corresponding to the genes not observed in post-PatH-Cap library from A shown in blue bars. Cumulative frequency of genes observed in the pre-PatH-Cap library shown in red

line. **D.** Iterative down-sampling of bacteria-aligned reads shows the number of genes observed and those detected with ≥10 unique transcripts at various sequencing depths for pre- and post-PatH-Cap libraries

Supplementary Figure S6. PatH-Cap enables analysis of low-input and single-cell dual RNA-seq libraries. A. Bacterial gene expression correlation of the sum of bulk populations pre- and post-PatH-Cap. **B**. Gene expression correlation of host genes in the sum of bulk libraries and the sum of all single cells pre-PatH-Cap from Fig 3B and 3E. **C.** Percent of aligned reads corresponding to *P. aeruginosa* mRNA in pre- and post-PatH-Cap libraries. Black dots are single cells, red dots are bulk populations (1,000 cells).

Supplementary Figure S7. Host steroid biosynthesis is not induced during *M. tuberculosis* infection. Normalized temporal expression (z-score) of host genes encoding proteins involved in cholesterol biosynthesis (*Fdft1*, *Msmo1*, *Sqle*, *Lss*, *Cyp51*, *Tm7*sf2, *Nsdhl*, *Hsd17b7*, *Ebp*, *Sc5d*, *Dhcr7*, *Dhcr24*).

Supplementary Figure S8. Temporal expression pattern of host genes encoding proteins related to intracellular iron regulation. Normalized temporal expression (Transcripts Per Million) of host genes encoding proteins involved in intracellular iron regulation (*Tfrc*, *Fth1*, *Ftl1*, *Slc11a1*, *Slc11a2, Slc40a1* and *Trf)* in infected (solid lines) and uninfected (dashed lines) macrophages.

SUPPLEMENTARY TABLE LEGENDS

Supplementary Table S1. Characterization of *P. aeruginosa* infection of bladder epithelial cells. Epithelial cells were infected with *P. aeruginosa* PAO1-GFP (3 biological samples) at MOI=25 for 1hr followed by the addition of gentamicin for 1hr. Cells were stained with DAPI and anti-Lamp-1 antibodies. Total number of uninfected and infected cells, and the number of bacterial per infected cell was determined by microscopy. Numbers in parenthesis indicate percent of all cells.

Supplementary Table S2. *P. aeruginosa* genes detected in all SC post-PatH-Cap libraries from infected cells are highly expressed in bulk populations. Twenty-three post-PatH-Cap SC libraries were sequenced >1X bacterial transcriptome coverage (>5,500 unique mRNA transcripts). Twenty-six genes were detected in all libraries. Rank expression (of 5,575 total bacterial genes) in bulk populations shown. *Based on Poulsen et al[23] where gene essentiality in 9 *P. aeruginos*a strains was determined in 5 media: LB, minimal medium (M9), synthetic cystic fibrosis sputum medium, urine and fetal bovine serum. Core essential: essential in all strains in all media. ND: essentiality could not be determined due to high sequence homology.

Supplementary Table S3. Cost analysis of PatH-Cap. Cost analysis for deep sequencing or PatH-Cap enrichment of 36 libraries (6 replicates at 6 time points) based on the median percentage bacterial mRNA obtained in our *M. tuberculosis* infection experiment in pre- and post-PatH-Cap libraries.

*Reads required per sample to obtain 3 million bacterial mRNA reads needed for comparative gene expression analysis as estimated by Haas et al[33].

**Sequencing on Novaseq S2: 4.1 billion reads $11,800

#Probes can be used for multiple experiments. Actual cost will decrease with increased number of experiments.

SUPPLEMENTARY DATASETS

Supplementary Dataset S1. Host and bacterial gene counts for pre- and post-PatH-Cap libraries from 1,000 *P. aeruginosa*-infected cells sorted 2 hours post-infection. Gene counts for 11 bulk libraries that passed quality filters as described in the Methods.

Supplementary Dataset S2. Host and bacterial gene counts for pre- and post-PatH-Cap libraries from single cells infected with *P. aeruginosa* 2 hours post-infection. Gene counts for 115 single cell libraries that passed quality filters as described in the Methods.

Supplementary Dataset S3. Host and bacterial gene counts for libraries from *M. tuberculosis* infected samples pre- and post-PatH-Cap. Gene counts for six infected ("WT") replicates and three uninfected ("Un") replicates at each of six time points (4, 24, 48, 72, 96 and 120 hours post infection).
